# Beneficial Effect of Exercise on Cognitive Function during Peripheral Arterial Disease: Potential Involvement of Myokines and Microglial Anti-Inflammatory Phenotype Enhancement

**DOI:** 10.3390/jcm8050653

**Published:** 2019-05-10

**Authors:** Marina Leardini-Tristao, Anne-Laure Charles, Anne Lejay, Mégane Pizzimenti, Alain Meyer, Vanessa Estato, Eduardo Tibiriçá, Emmanuel Andres, Bernard Geny

**Affiliations:** 1 Laboratório de Imunofarmacologia, Instituto Oswaldo Cruz, FIOCRUZ, Avenida Brasil 4365, Rio de Janeiro 21040-360, Brazil; tristaomarina@gmail.com (M.L.-T.); estato@ioc.fiocruz.br (V.E.); 2 Université de Strasbourg, Fédération de Médecine Translationnelle de Strasbourg (FMTS), Faculté de Médecine, Equipe d’Accueil 3072, 11 Rue Humann, 67000 Strasbourg, France; anne.laure.charles@unistra.fr (A.-L.C.); anne.lejay@chru-strasbourg.fr (A.L.); megane.pizzimenti@hotmail.fr (M.P.); alain.meyer1@chru-strasbourg.fr (A.M.); 3 Service de Chirurgie Cardiovasculaire, Pôle de Pathologie Cardiaque, Hôpitaux Universitaires, CHRU Strasbourg, 67000 CEDEX, France; 4 Service de Physiologie et d’explorations Fonctionnelles, Hôpitaux Universitaires de Strasbourg, Nouvel Hôpital Civil, 1 place de l’Hôpital, 67091 Strasbourg CEDEX, France; 5 Instituto Nacional de Cardiologia, Ministério da Saúde, Rua das lanjeiras 374, Rio de Janeiro 22240-006, Brazil; etibi@uol.com.br; 6 Service de Médecine Interne, Diabète et Maladies Métaboliques, Pôle M.I.R.N.E.D., Hôpitaux Universitaires, CHRU Strasbourg, 67000 CEDEX, France; emmanuel.andres@chru-strasbourg.fr

**Keywords:** peripheral arterial disease, ankle-brachial index, cognitive dysfunction, brain, exercise, myokines, microglia, BDNF, cathepsin-B, irisin

## Abstract

Peripheral arterial disease (PAD), leading to intermittent claudication, critical ischemia with rest pain, and/or tissue damage, is a public health issue associated with significant morbidity and mortality. Little is known about the link between PAD, cognitive function, and whether exercise might reduce cognitive dysfunction in PAD patients, as previously observed concerning both quality of life and prognosis. This review highlights the fact that patients suffering from PAD often demonstrate cognitive dysfunction characterized by reduced performance in nonverbal reasoning, reduced verbal fluency, and decreased information processing speed and a greater risk for progression toward dementia. Further, the data presented support that physical exercise, likely through myokine secretion and microglial anti-inflammatory phenotype enhancement, might participate in the cognition protection in common clinical settings.

## 1. Introduction

Peripheral arterial disease (PAD) is a public health issue resulting in significant morbidity and mortality. Its prevalence ranges from 4 to 8% in Europe, reaching 13% in 70-year-old patients. First classified by Fontaine and Leriche in four stages, asymptomatic PAD (stage I); mild claudication (stage II a); moderate to severe claudication (stage II b); ischemic rest pain (stage III); and ulceration or gangrene (stage IV); PAD is now classified in intermittent claudication and critical ischemia with rest pain and/or tissue damage [1,2,3,4,5]. To avoid local damage which might lead to limb amputation and to reduce death occurrence, several therapeutic approaches have been tested, including exercise. Indeed, low exercise capacity is significantly associated with severe comorbidities [6] and exercise improved both local and general prognosis in patients suffering from PAD, enhancing walking ability and preventing serious cardiovascular complications [7,8,9,10]. At a cellular level, the beneficial effects of exercise can be explained by improvements in muscle biogenesis, mitochondrial function, and energy balance [11]. Even in the case of chronic critical limb ischemia, experimental data support that moderate exercise can reduce skeletal muscle damage, improving functional scores, restoring mitochondrial respiration and calcium retention capacity, and enhancing anti-oxidant defenses, such as superoxide dismutase 1 and 2, and catalase [12]. 

Therefore, exercise, either performed in hospital or out of hospital facilities, is a widely accepted therapy, ameliorating muscle function and more generally PAD patient’s quality of life [13]. 

To date, limited data have been reported concerning the relationship between PAD and brain function and whether exercise might improve cognition in patients with PAD. However, evidence supports that several types of exercise improve cognitive performance in humans [14,15]. 

The aim of this review is to highlight the fact that patients suffering from PAD often demonstrate with cognitive dysfunction and that exercise, likely through myokine secretion and microglial anti-inflammatory phenotype enhancement, might help to protect the brain function in common clinical settings.

## 2. Cognitive Function in Patients with Peripheral Arterial Disease

**Figure 1:**
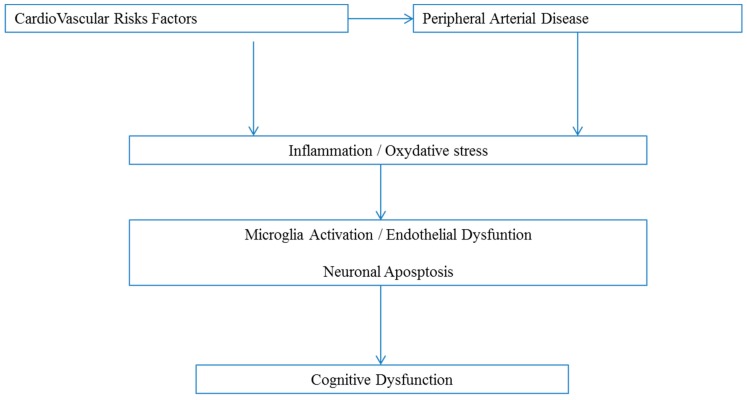
Mechanisms leading to cognitive dysfunction in peripheral arterial disease (PAD) patients.

PAD patients are often viewed as presenting with cognitive dysfunction. Such impaired cognitive processes are not unexpected since both diseases share common cardiovascular risk factors, such as hypertension, diabetes, hypercholesterolemia, obesity, sedentary lifestyle, and smoking [5,16,17,18,19]. More specifically, inflammation, oxidative stress, mitochondrial, and vascular dysfunction are key factors in the pathophysiology of both PAD and neurodegenerative diseases [12,20,21,22]. Thus, although an experimental study failed to demonstrated brain mitochondrial dysfunction in the setting of aortic clamping [23], peripheral inflammatory factors released during the ischemic process have been shown to impair several remote organs, including the brain [24,25] (Figure 1).

### 2.1. Main Clinical Data

Recently, confirming experimental data, clinical trials have shown the presence of cerebrovascular disease in the context of PAD [26,27,28,29]. Thus, compared to hypertensives and normotensives patients, patients with PAD had significantly lower performance on seven tests of nonverbal memory, concentration, executive function, perception-motor speed, and manual dexterity. However, PAD patients performed better than stroke patients. These results were independent of age, education, and depression scores. PAD patients with higher plasma glucose levels and diastolic blood pressure had a worse performance in cognitive tests [27]. The findings also suggest that the degree of cognitive dysfunction is associated with increasingly severe indicators of cardiovascular disease. 

In 2006, a study investigated the cognitive function of patients with asymptomatic peripheral arterial disease, without the occurrence of stroke or transient ischemic attacks. The results showed that the patients scored worse than healthy controls on five cognitive tests (digit span backward, trail making A and B, and Rey–Osterrieth complex figure copy and delayed recall), related to attention, verbal working memory, perceptuo-motor speed, visuo-constructive skills, and visual memory [30]. 

Cognitive function was also studied in patients with PAD or diabetes mellitus who underwent lower limb amputation. Patients were investigated before amputation and 6 weeks and 4 months after amputation. No significant differences were found in neuropsychological tests between 6 weeks and 4 months after amputation surgery. However, the patients presented an improvement in cognition when compared to the results before surgery. The authors suggested that this improvement was associated with the perceived increase in general health [31]. Another study with patients amputated due to peripheral vascular disease demonstrated that they performed significantly worse than controls in certain measures of psychomotor speed and problem-solving or abstract reasoning. The authors hypothesized that cognitive deficits may be the result of unrecognized concomitant cerebrovascular diseases, which are part of a generalized pattern of vascular disease [32]. 

In a study conducted by Gardner et al. [28], patients with PAD were grouped according to their performance on a mini-mental state examination (MMSE). Patients who scored at least 28 out of 30 points on MMSE underwent an assessment of several aspects, including peak walking time and health-related quality of life. The group with lower MMSE scores had a lower education level, a greater prevalence of coronary artery disease, chronic obstructive pulmonary disease, and arthritis, and took more medications for diabetes. The mean peak walking time was significantly reduced in the group with lower MMSE scores compared to the group with higher MMSE scores. The group with lower MMSE scores perceived less ability to perform the high-intensity exercise of climbing stairs, and they had lower levels of health-related quality of life (Table 1).

### 2.2. The Ankle-Brachial Index as an Indicator of Cognitive Dysfunction in PAD

The ankle-brachial index (ABI) is very useful in the evaluation of patients with suspected PAD. The test is based on the systolic pressure measurement from the posterior tibial arteries and dorsalis pedis of each leg, normalized by the highest brachial pressure of each arm to assess the ankle-brachial index. The typical cutoff point for diagnosing PAD is ≤0.90 at rest [4]. According to Hart et al., the ABI was significantly correlated with intramuscular inflammation, oxidative stress, and mitochondrial reactive oxygen species (ROS) generation [33]. 

**Figure 2:**
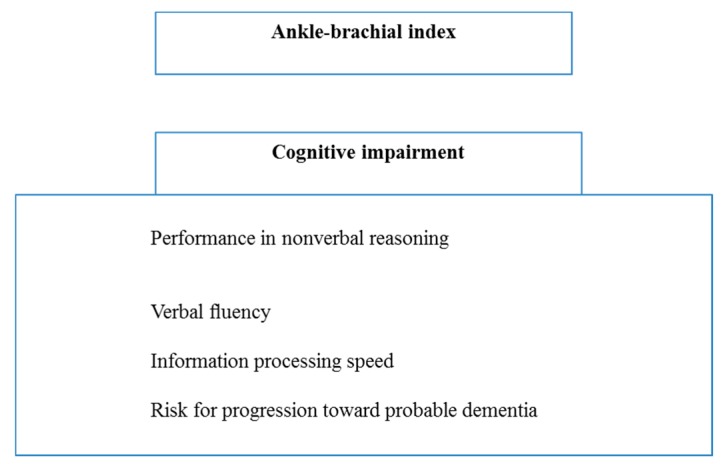
Ankle-brachial index is an indicator not only for PAD, but also for cognitive dysfunction.

Interestingly, in a cohort study [34], 717 men and women (aged 55 to 74) from the general population were followed for 10 years. The results indicated that the lower the ABI, the worse the performances in nonverbal reasoning, verbal fluency, and information processing speed. These data strongly support that ABI is predictive for cognitive impairment. Accordingly, a relationship between ABI and cognitive function was found in a cross-sectional survey in elderly Chinese people, after logistic regression and adjustment for age and sex. The presence of cardiovascular diseases (stroke, hypertension, myocardial infarction) and cognitive impairment was positively associated with low ABI [35]. Finally, Espeland et al. demonstrated that in an older cohort of non-demented sedentary individuals, lower ABI was independently correlated with cognitive function and associated with greater 2-year risk for progression to mild cognitive impairment or probable dementia [36]. This association between ABI and cognitive impairment was confirmed in a cross-sectional study where aging resulted in a higher proportion of individuals with low ABI or cognitive impairment [37]. Further, individuals with low ABI had a higher frequency of cerebral lacunar infarcts, intracranial stenosis, cortical thinning, and lower verbal memory performance [38]. In a systematic review and meta-analysis, ABI proved to be an accessible tool for patients with stroke, demonstrating an association between low ABI (≤0.9) and recurrent stroke and future vascular events [39]. Thus, originally proposed for the noninvasive diagnosis of PAD, ABI might be a simple measure to assess whether PAD patients would be more likely to develop cognitive impairments. Together, these results suggest that ABI is an indicator not only of PAD, but also of cognitive function (Figure 2) and ABI determination might further support exercise as a therapeutic approach.

## 3. Implications of Myokines in the Beneficial Effects of Exercise on Brain Function in Peripheral Arterial Disease Patients

The regular practice of physical exercise is one of the most effective treatments for patients with intermittent claudication [40]. Physical exercise is a good and low-cost non-pharmacological therapeutic strategy, minimizing the effects of ischemia-reperfusion injury and improving the clinical and mental state of the patients. Among the positive effects of physical exercise are the reduction of inflammatory factors and oxidative stress, increased angiogenesis, and improvement of endothelial and cognitive function. According to Cavalcante et al. [29], cognitive performance in patients with PAD is positively associated with walking ability and moderate to vigorous intensity physical activity.

Skeletal muscle accounts for approximately 40% of the total body mass and evidence indicates that, besides its locomotor function, skeletal muscle secretes cytokines or other peptides that may exert paracrine, or endocrine effects in distant organs [41,42,43]. Such myokine secretion is generally enhanced by exercise and they may play important anti-inflammatory, neuroprotective and neurogenic roles [43,44,45]. Additionally, a preferential shift toward a microglial anti-inflammatory phenotype likely participates in exercise-induced protection of the cognitive function in PAD patients.

### 3.1. Involvement of Myokines in Exercise-Induced Protection of the Cognitive Function 

Studies have shown that anti-inflammatory cytokines, such as IL-6, IL-1RA, and IL-10, are increased after exercise. Physical exercise also induces the expression of brain-derived neurotrophic factor (BDNF) mRNA in the hippocampus, an important factor responsible for neuron survival, maturation, proliferation, and plasticity, thus playing a significant role in learning and memory [46,47]. 

Moon et al. [48] suggested that cathepsin-B may be an important mediator of the effects of physical exercise on cognition. After 12 weeks of exercise, cathepsin-B levels increased in mice plasma and muscle, and the cathepsin-B treatment in neuronal cells increased BDNF expression. Further, cathepsin-B knockout mice showed no improvement in neurogenesis and spatial memory, as compared to control mice. Importantly, similar results were found in rhesus monkey and humans, correlating the increase of cathepsin-B plasma levels with cognition improvement [48]. 

Irisin appears also as an exercise-induced myokine, released upon the cleavage of the membrane-bound precursor protein fibronectin type III domain-containing protein 5 (FNDC5). It is associated with homeostasis processes such as glucose metabolism, insulin sensitivity, and fat browning [49]. Recently, irisin demonstrated an important role as a neurotrophic factor promoting survival, maintenance, and function of neuronal cells [50]. Wang et al. [51], showed that irisin protected the neurons, reduced the release of IL-1β and IL-6, and reduced the expression of cyclooxygenase 2 (proinflammatory factor) in cultured astrocytes exposed to β-amyloid. Accordingly, Lourenco et al. [52] showed that an intra-cerebroventricular infusion of amyloid-β oligomers (AβOs) induced significant reductions of FNDC5 mRNA expression in mice hippocampus. Similarly, impaired maintenance of hippocampal long-term potentiation and memory were observed in knocked down FNDC5/irisin mice. However, when AβOs mice received an intravenous injection of FNDC5, hippocampus FNDC5 levels increased and memory was protected. Reduced levels of FNDC5/irisin in the brain and cerebrospinal fluid in patients with Alzheimer’s disease further support the implication of such defective signaling in humans.

Concerning exercise/irisin relationships, some controversies still exist. Physical exercise protected AβOs mice from cognitive dysfunction and increased the hippocampal levels of FNDC5/irisin, as compared to sedentary mice [52]. Consistently, mice performing physical exercise for 2 weeks prior to cerebral ischemia demonstrated better scores on a neurological function test. Further supporting the neuroprotective effects of irisin, such beneficial effects of physical exercise were abolished when blocking irisin one hour before cerebral ischemia [53].

However, since the original study by Bostrom et al. showing that exercise increases irisin in mice and humans [54], others proposed negative evidence of a possible expression and concentrations of irisin in skeletal muscles and serum during exercise or training [55,56] and the physiological role of FNDC5/irisin in mediating responses to exercise was challenged [57]. Nonetheless, several publications supported exercise-related changes in irisin [58]. For example, an exercise-related increase in irisin resulted in BDNF expression in the hippocampus [59] and Küster et al. [60] observed a positive correlation between irisin, BDNF levels, and cognition in older adults at risk of dementia after 10 weeks of physical exercise. Accordingly, physical exercise-induced cognitive improvement together with increased neurogenesis and BDNF, FNDC5 levels, and synaptic proteins in the hippocampus of 5XFAD transgenic mice, a model for Alzheimer’s disease [61]. It is therefore likely that the high expression of the irisin precursor FNDC5 in the brain, including in the cerebellar Purkinje cells, the hypothalamus and hippocampus might participate in the beneficial effects of exercise on brain and neurodegenerative diseases [62].

The type of exercise might modulate irisin /brain interactions. Irisin appeared to not be involved in resistance training because it failed to activate PGC-1α1, a transcription regulator upstream of the FNDC5 [62]. The timing of blood withdrawal might also be essential since elevated irisin has been shown to be transient during exercise in young men and women [63]. This might also explain some discrepant data previously reported [53,54,55,56,57,58,59,60,61,62,63]. Ongoing identification of irisin receptors and detailed signaling pathways triggered during exercise will be useful to increase our knowledge and allow us to optimize irisin activity modulation.

Interleukin 6 (IL-6) has been described as a double-edged sword, which may present pro- or anti-inflammatory effects, and consequently neurodegenerative or protective actions [64,65]. Mainly defined as a proinflammatory cytokine, IL-6 was the first myokine found to be secreted into the bloodstream in response to muscle contractions, with a considerable increase in plasma up to 100-fold in response to exercise [66,67]. 

The role of IL-6 in the brain is rather complex. IL-6 differentially influences microglia, mediating neuroprotective [68] and neurotoxic microglial responses [69]. It exerts neurotrophic actions and is also a mediator of acute inflammation [70]. A number of studies have reported elevated levels of IL-6 during central nervous system (CNS) disorders [65], but on the other hand Ma and co-workers [71], demonstrated that IL-6 reduced neuronal cytosolic Ca^2+^ overload, mitochondrial membrane depolarization, and neuronal induced by NMDA, demonstrating that IL-6 has a neuroprotective property. Similarly, the IFN-γ and IL-6 in an acute neuro-inflammatory environment are neuroprotective via ERK and/or STAT3 [72]. Gmiat et al. found that a single bout of high-intensity circuit training was able to improve concentration and spatial memory in young women, and that irisin and IL-6 releases from exercise (respecting intensity and periods of adequate rest and nutrition) might be considered as a sensor stimulating BDNF synthesis [73]. Starkie et al. demonstrated that exercise and IL-6 infusion inhibit TNF-α production, suggesting that IL-6 might mediate anti-inflammatory activity during exercise [74]. 

**Figure 3:**
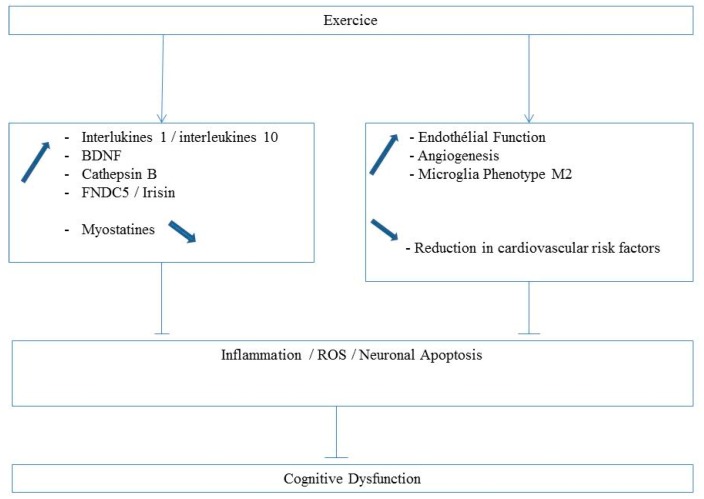
Mechanisms likely involved in exercise-related brain protection in PAD patients.

Myostatin, another important myokine, is a member of the TGF-β superfamily, and its inactivation induces skeletal muscle hypertrophy in mice and humans. Myostatin also regulates the maintenance of metabolic homeostasis and modulation of adipose tissue function and mass [75,76,77]. Myostatin could be expressed in different brain regions, but its function in the brain is still unknown [75]. Interestingly, Lin et al. [78] demonstrated that myostatin levels were elevated in the gastrocnemius muscle and that the extent of muscle mass loss was associated with the severity of cognitive deficits in transgenic mice mimicking Alzheimer’s disease. Myostatin knockdown in the gastrocnemius increased grip strength and muscle mass and improved memory in such transgenic mice. The authors concluded that cognitive dysfunction may be mediated or triggered by myostatin expression and suggested that myokine modulation may be a therapeutic intervention against muscular and cerebral dysfunctions (Figure 3).

### 3.2. Microglial Anti-Inflammatory Phenotype and Exercise

Recently, another topic has been attracting interest in the scientific community. It is the study of the microglial anti-inflammatory phenotype. Microglia is a highly plastic group of immune cells that reside in the CNS. The classic microglia activation is pro-inflammatory, characterized by the release of TNF-α and IL-1β, inducing neuro-inflammation and reduction in hippocampal neurogenesis [79,80,81]. However, some studies suggest that microglia is also capable of repairing tissue damage by producing anti-inflammatory cytokines, such as TGF-β; growth factors, such as IGF-1 and BDNF [81]. The biphasic pro or anti-inflammatory role of microglia is dictated by the type of expressed phenotype. During the pro-inflammatory response, microglia expresses the M1 phenotype, while in the anti-inflammatory response the M2 phenotype is expressed. This biphasic response of microglia is linked to other cerebral cells, including astrocytes, oligodendrocytes, and neurons [82]. 

Beneficial effects of voluntary running in aged mice were related to the induction of a neuroprotective phenotype in microglia; and the proportion of BDNF positively co-labeled with microglia was correlated with the number of neurons [83]. Similar results were found by Kohman et al. [84], demonstrating that running increased the proportion of microglia expressing IGF-1 and the survival of neurons, suggesting that exercise shifts toward microglia pro-neurogenic phenotype allowing neuroprotection. Further, in a chronic cerebral hypoperfusion model, 28 days of exercise transformed the microglial phenotype from M1 to M2, improving the cognitive function in rats [85]. Further supporting the beneficial effect of exercise on microglia, a recent study showed that 4 weeks of physical exercise reduced the M1 phenotypic markers (CD32, CD86, and iNOS) and increased the M2 phenotypic markers (ARG1, TGF-β, and CD206) in the hippocampal microglia of an Alzheimer’s disease model. This was accompanied by a reduction in inflammatory factors, such as IL-1β and TNF-α, and by an increase in anti-inflammatory factors, such as IL-4 and IL-10. In addition, exercise reduced neuronal loss, oxidative stress, apoptosis, and pro-apoptotic cascade in the hippocampus. Exercise also improved the cognition, as inferred by better scores in three different tasks (Barnes maze task, passive avoidance test, and novel object recognition test) [86]. Similarly, intravenous administration of irisin 30 min after cerebral ischemia inhibited the activation of microglia, oxidative stress, and neuroinflammation in mice [53]. Finally, environmental enrichment with toys, tunnels, and running wheels, allowed the analysis of the importance of microglia in the neurogenesis of adult hippocampus. The activation of microglia was associated with hippocampal neurogenesis after 6 weeks of environmental enrichment [87]. Taken together, these studies suggest that physical exercise might modulate the activation of microglia with a protective impact on neurogenesis.

## 4. Exercise Characteristics and Improvement in Cognitive Function

Which kind of exercise might be the most beneficial in order to improve the cognitive function remains to be investigated in PAD. The effects of exercise training on intermittent claudication have been recently reviewed and, clearly, physical exercise either supervised and/or home-based improved PAD patient performance and quality of life. Several types of exercise were beneficial but exercising 3-5 times a week for 30–50 min was often used and demonstrated increased walking time without pain and increased exercise capacity [88,89]. In a study including 114 PAD patients, 3 types of physical exercise, supervised physical exercise, "home-based", and resistance programs, performed 3 times a week for 12 weeks were investigated. Even home-based exercise training improved circulating markers of antioxidant capacity, angiogenesis (VEGF), endothelium-derived inflammation (E-selectin), and blood glucose concentration [90]. Regarding intensity, a body of evidence suggests that higher exercise intensities may maximize the health outcomes of PAD patients [91]. Accordingly, a systematic review and meta-analysis supported that patients with PAD tolerated vigorous exercise intensity and intermittent aerobic exercise, the intensity being based on the occurrence of claudicating pain. Exercise duration of 24 weeks generated positive effects, the better exercise-induced responses being obtained in patients with mild pain [92].

Thus, one might propose that exercise protocols that are effective on PAD symptoms should also be effective on cerebral functions, following a dose–response relationship. Indeed, in patients with known cardiovascular diseases such as heart failure and stroke, the degree of improvement in cognitive performance was related to exercise duration, frequency, and intensity. Further, combined interventions might be more efficient than endurance and resistance training alone [93].

Interestingly, although irisin increased similarly in young men and women during exercise [63], we are not aware of studies investigating specifically potential gender differences in the relationship between PAD, exercise, and cognitive function. Nevertheless, a recent work stressed the role of gender in memory function, suggesting that exercise protocol should be further personalized [94].

## 5. Conclusion

In conclusion, PAD and ischemia-reperfusion damage are not restricted to the lower limbs, and significant deleterious effects can be observed in the brain. Particularly, PAD patients often present with cognitive dysfunctions, related to their PAD degree as inferred from the relationship between the ankle brachial index and the cognition level. The mechanisms and pathways that link the periphery and the CNS are still under investigation, but oxidative stress and inflammation appear to play key roles.

Physical exercise can already be considered as an efficient therapeutic option, reducing PAD-related cognitive dysfunctions, potentially through exercise-induced myokine secretion and microglial anti-inflammatory phenotype enhancement. Reduction in cardiovascular risk factor, improved endothelial function, decreased inflammation and oxidative stress should also participate in the beneficial effects of exercise in patients suffering from PAD.

## Figures and Tables

**Table 1:** Cognitive function in PAD patients.

Reference	Study Sample	Total Sample Size and SEX	Mean Age	Cognitive Measures	Cognitive Results
Phillips et al., 1993 [32] (Arch Phys Med Rehabil.)	Patients with lower-extremity amputations secondary to PAD and healthy volunteers	37: PAD group (4 women and 10 men) and healthy volunteers (9 women and 5 men)	67.4 ± 14.8 and 69.9 ± 9.3	Learning and memory (WAIS-R Digit Symbol subtest, WMS-R); language and verbal ability; visuoperceptual organization and constructional abilities; problem solving (MCST), abstract reasoning, and concept formation; social judgement and sequential reasoning; psychomotor function.	The PAD amputee patients performed more poorly than controls (*p* < 0.002, one-tailed) on the WAIS-R Digit Symbol subtest and obtained fewer categories on the MSCT than did the controls. There were trends (*p* < 0.01, one-tailed) toward lower patient scores on a number of other neuropsychological tests including the WAIS-R Vocabulary, Arithmetic, Similarities, and picture arrangement subtests, oral fluency (COWAT, orthographic condition), and the copy administration of the ROCF.
Waldstein et al., 2003 [27] (Psychosomatic Medicine)	PAD, stroke, hypertensive and normotensive patients	107: Normotensive group (7 women and 16 men), hypertensive group (5 women and 15 men), PAD group (10 women and 28 men) and stroke group (6 women and 20 men)	66.3 ± 5.8 70.0 ± 5.7 69.8 ± 7.0 62.3 ± 8.1	Tests for verbal memory (WMS-R) non verbal memory attention, was evaluated by recall of geometric figures using the Visual Reproductions subscale of the WMS-R. Trail Making Test Parts A and B and the Stroop Color-Word Test for perceptuo-motor speed and executive functions. Motor speed and manual dexterity were examined with the Grooved Pegboard test.	PAD patients performed more poorly than normotensive patients in tests of non verbal memory, verbal working memory (*p* < 0.002), perceptuomotor speed, attention and mental flexibility and motor speed and manual dexterity (*p* < 0.00001) and compared to hypertensive patients in verbal memory (*p* < 0.002), verbal working memory perceptuomotor speed, attention and mental flexibility. Stroke<PAD<Hypertensive<Normotensive
Mangiafico et al., 2006 [30] (Age and Ageing).	Asymptomatic PAD (APAD) - stage I	328: APAD group (42 women and 122 men) and Control group (44 women and 120 men)	70.0 ± 3.4 and 70.3 ± 3.7	Cognitive domains of attention and verbal working memory (Digit Span Forward and Backward), perceptuomotor speed, attention and mental flexibility (Trail Making Test), visuoconstructive skills and visual memory ROCF Copy and ROCF Delayed Recall and the global cognitive functioning (MMSE).	Patients with APAD scored significantly worse (*p* < 0.0001) than control subjects on five cognitive tests: Digit Span Backward, Trail Making A, Trail Making B, ROCF Copy and ROCF Delayed Recall
Williams et al., 2014 [31] (Arch Phys Med Rehab.)	PAD or DM patients with lower extremity amputation.	87: Presurgicaly (1 woman and 28 men) and postsurgicaly (6 women and 52 men)	63 ± 10 and 62 ± 8	Neuropsychological Test Score: executive function (semantic fluency), auditory-verbal learning (list learning), and verbal memory (list recall)	Improvement in overall performance between presurgery and 6 weeks (*p* = 0.03) and presurgery and 4 months (*p* = 0.06), but no differences between 6 weeks and 4 months after amputation.
Gardner et al., 2016 [28] (Journal of Vascular Surgery)	Symptomatic PAD: Patients with a perfect MMSE score of 30 points and patients with score < 30 points.	246: PAD patients with score of 30 (65 women and 58 men) and PAD patients with score <30 (61 women and 62 men)	64 ± 10 and 65 ± 11	MMSE questionnaire	Lower cognitive screening scores were associated with greater ambulatory impairment. Worse cognitive status was associated with lower scores in multiple dimensions of health-related QoL; The group with lower MMSE scores had a lower education level (*p* < 0.01), a greater prevalence of CAD (*p* = 0.02), (*p* = 0.01), and arthritis (*p* < 0.01), and took more medications for diabetes (*p* < 0.01)
Cavalcante et al., 2018 [29] (Eur. J. Vasc. Endovasc. Surg.)	Symptomatic PAD (intermittent claudication in one or two legs, stage)	130: 29 women and 101 men	67 ± 8	Cognitive function; global, memory, executive function and attention by MoCA test	86% of patients were classified as probably having a cognitive impairment; Greater memory performance was associated with greater moderate to vigorous physical activity leaves (*p* = 0.044) and walking capacity (*p* = 0.033)

CAD: coronary artery disease; COPD: chronic obstructive pulmonary disease; COWAT: Controlled Oral Word Association Test; DM: diabetes mellitus; MCST: Modified Card Sorting Test; MMSE: mini-mental state examination questionnaire; MoCA: Montreal cognitie assessment; PAD: peripheral arterial disease; QoL: health-related quality of life; ROCF: Rey–Osterrieth Complex Figure SPMSQ: short portable mental status questionnaire; WAIS-R: Wechsler Adult Intelligence Scale-Revised; WMS-R: Wechsler Memory Scale-Revised.
